# Risk factors for deep vein thrombosis in patients with pelvic or lower-extremity fractures in the emergency intensive care unit

**DOI:** 10.3389/fsurg.2023.1115920

**Published:** 2023-03-30

**Authors:** Dongcheng Shi, Bingbo Bao, Xianyou Zheng, Haifeng Wei, Tianhao Zhu, Yi Zhang, Gang Zhao

**Affiliations:** ^1^ Department of Emergency Medicine, Shanghai Sixth People's Hospital Affiliated to Shanghai Jiao Tong University School of Medicine, Shanghai, China; ^2^ Department of Orthopedic Surgery, Shanghai Sixth People's Hospital Affiliated to Shanghai Jiao Tong University School of Medicine, Shanghai, China

**Keywords:** Autar deep vein thrombosis scale, deep vein thrombosis, lower-extremity fracture, pelvic fractures, trauma

## Abstract

**Introduction:**

This study aimed to investigate the incidence of deep vein thrombosis (DVT) in patients with pelvic or lower-extremity fractures in the emergency intensive care unit (EICU), explore the independent risk factors for DVT, and investigate the predictive value of the Autar scale for DVT in these patients.

**Methods:**

The clinical data of patients with single fractures of the pelvis, femur, or tibia in the EICU from August 2016 to August 2019 were retrospectively examined. The incidence of DVT was statistically analyzed. Logistic regression was used to analyze the independent risk factors for DVT in these patients. The receiver-operating characteristic (ROC) curve was used to evaluate the predictive value of the Autar scale for the risk of DVT.

**Results:**

A total of 817 patients were enrolled in this study; of these, 142 (17.38%) had DVT. Significant differences were found in the incidence of DVT among the pelvic fractures, femoral fractures, and tibial fractures (*P* < 0.001). The multivariate logistic regression analysis showed multiple injuries (OR = 2.210, 95% CI: 1.166–4.187, *P* = 0.015), fracture site (compared with tibia fracture group, femur fracture group OR = 4.839, 95% CI: 2.688–8.711, *P* < 0.001; pelvic fracture group OR = 2.210, 95% CI: 1.225–3.988, *P* = 0.008), and Autar score (OR = 1.198, 95% CI: 1.016–1.353, *P* = 0.004) were independent risk factors for DVT in patients with pelvic or lower-extremity fractures in the EICU. The area under the ROC curve (AUROC) of the Autar score for predicting DVT was 0.606. When the Autar score was set as the cutoff value of 15.5, the sensitivity and specificity for predicting DVT in patients with pelvic or lower-extremity fractures were 45.1% and 70.7%, respectively.

**Discussion:**

Fracture is a high-risk factor for DVT. Patients with a femoral fracture or multiple injuries have a higher risk of DVT. In the case of no contraindications, DVT prevention measures should be taken for patients with pelvic or lower-extremity fractures. Autar scale has a certain predictive value for the occurrence of DVT in patients with pelvic or lower-extremity fractures, but it is not ideal.

## 1. Introduction

Venous thromboembolism (VTE) (1) refers to a disease in which abnormal blood clots in veins block the venous lumen and lead to venous blood reflux disorders, mainly including deep vein thrombosis (DVT) and pulmonary thromboembolism (PE). DVT is a common complication after traumatic injury (2), especially in patients with lower-extremity fractures (3, 4). The acute phase of DVT of lower extremities may lead to swelling and pain of lower extremities, weakened or disappeared pulsing of the dorsal foot artery, severe systemic reaction, shock, and venous jaundice if not treated in time (5). The chronic phase can develop into post-thrombotic syndrome, with clinical manifestations of chronic lower-extremity venous insufficiency, including heaviness, distending pain, varicose veins, skin pruritus, pigmentation, and so forth, and high swelling and ulceration of the lower extremity in severe cases (6). Once the thrombus attached to the venous wall falls off, it can drift with the blood flow. If it blocks the main pulmonary artery or branch, it can lead to PE, cause chest pain and other clinical manifestations (7), and even endanger the patient's life (8, 9). Some studies have shown that PE is an important cause of sudden death in hospitalized patients (7, 10). In the absence of DVT prevention measures, 40%–70% of patients with fractures develop DVT during the perioperative period (11). The incidence of DVT in patients with pelvic fracture was 5%–21.09% (12, 13), the incidence of DVT in patients with femoral fracture was 6.85%–32% (14, 15), and the incidence of DVT in patients with tibial fracture was 2.09%–16.3% (14, 16) after the use of low-molecular-weight heparin (LMWH) and other preventive measures.

Although LMWH has a good preventive effect on DVT (17), some patients with fractures still inevitably develop DVT. Compared with orthopedic wards, patients with pelvic or lower-extremity fractures in the emergency intensive care unit (EICU) are more critical and complex, often complicated with multiple injuries, sepsis, hemorrhagic shock, and other potentially life-threatening conditions. Indwelling deep venous catheters for timely rescue measures may damage the venous wall. Also, prolonged bed rest and the use of sedative muscle relaxants may aggravate the slow venous flow, leading to a higher risk of DVT in patients with fractures in the EICU than in patients with fractures in orthopedic wards. Some patients with DVT do not have lower-limb swelling and pain. Also, patients with fracture can easily be confused with DVT symptoms due to limb swelling and pain caused by trauma, immobilization, and surgery. Hence, it is not reliable for clinicians to judge DVT simply by experience. Establishing a DVT risk assessment scale by medical history, trauma, and other indicators is necessary to quantify the risk of DVT in patients with fractures. At present, various scales have been used to assess the risk of DVT in trauma patients, among which multiple variables (such as: age, physical activity, type of trauma, etc.) in the Autar deep vein thrombosis scale (Autar scale) have been shown to have an important impact on DVT (18), which appears to predict risk of DVT in patients with fractures. This study focuses on the incidence of DVT in patients with pelvic or lower extremity fractures in EICU and the predictive value of Autar scale for DVT in patients with pelvic or lower extremity fractures, and at the same time explores the independent risk factors for DVT.

## 2. Materials and methods

### 2.1. Participants

This study was retrospective in nature. Patients hospitalized in the EICU of the Sixth People's Hospital Affiliated to Shanghai Jiao Tong University from August 2016 to August 2019 were enrolled, and their clinical data were analyzed. The inclusion criteria were as follows: (1) patients with fractures of the pelvis, femur, or tibia; (2) age ≥18 years; (3) less than 2 weeks from injury to the surgery; and (4) lower-extremity compression ultrasound (CUS) performed before 2 days internal fixation. The exclusion criteria were as follows: (1) pathological fracture; (2) two or more fracture sites present at the same time; (3) anticoagulation or antiplatelet therapy before fracture; (4) tumors or hematological diseases; (5) DVT present before the fracture; (6) patient in gestation; and (7) incomplete clinical data. Except for patients with brain trauma, all participants received LMWH once daily subcutaneously to prevent DVT. Brain trauma included brain contusion, traumatic cerebral hemorrhage, subarachnoid hemorrhage, subdural hemorrhage, and epidural hemorrhage. This study was approved by the ethics committee of the Sixth People's Hospital Affiliated to Shanghai Jiao Tong University [Approval No. 2022-KY-010 (K)].

### 2.2. Study design

The clinical data of patients were extracted *via* the electronic medical record system of the hospital information database, including (1) demographic data; (2) diagnosis information; (3) acute physiology and chronic health evaluation II (APACHE II), injury severity score (ISS), Glasgow coma scale (GCS), sequential organ failure assessment, and Autar score based on Autar scale 2002 within 24 h of hospitalization (19); (4) vital signs on admission; (5) laboratory data within 24 h of hospitalization; and (6) CUS examination results of the lower extremity. The participants were divided into a DVT group and a non-DVT group based on the results of the lower-extremity CUS examination. A CUS examination is performed by a professional ultrasound doctor to determine whether DVT is formed; the criteria for CUS diagnosis of DVT include: solid echo filling in the blood vessel, the lumen cannot be compressed by the probe, and the color blood flow signal is bypassed or there is no blood flow signal.

Multiple injuries are defined as follows (20): ISS score of trauma patients >15 and abbreviated injury scale score of at least two parts of the body ≥3, plus at least one of the following five pathological conditions: (1) hypotension (systolic blood pressure ≤90 mm Hg); (2) unconsciousness (GCS score ≤ 8); (3) acidosis (alkali residue ≤–6.0 mmol/L); (4) coagulation dysfunction (international standardized ratio ≥1.4 or partial thromboplastin time ≥40 s); and (5) age ≥70 years. Due to patients with fractures are high-risk groups for DVT, emergency physicians in EICU routinely performed Autar scores upon admission and recorded them in the medical record system.

### 2.3. Statistical analysis

Measurement data with normal distribution were expressed as mean ± standard deviation (*x¯ *± *s*), and a comparison between the two groups was performed using the independent-sample *t* test. Measurement data with non-normal distribution were expressed as median (quartile) [*M*(*Q_L_, Q_U_*)], and a comparison between the two groups was performed using the Mann–Whitney *U* test. The corrected chi-square test was used when the theoretical frequency was less than 5. The Fisher exact probability method was used when the theoretical frequency was less than 1. The SPSS Statistics 19.0 software was used for data processing and statistical analysis. The univariate and multivariate logistic regression analyses were performed for the indicators with significant differences between the two groups to find the independent risk factors for DVT in patients with pelvic or lower-extremity fractures. By drawing the receiver-operating characteristic (ROC) curve, the area under the ROC curve (AUROC) and the Youden index were used to evaluate the predictive value of the Autar score for DVT. A *P* value <0.05 indicated a statistically significant difference.

## 3. Results

### 3.1. Characteristics of study participants

**Figure 1:**
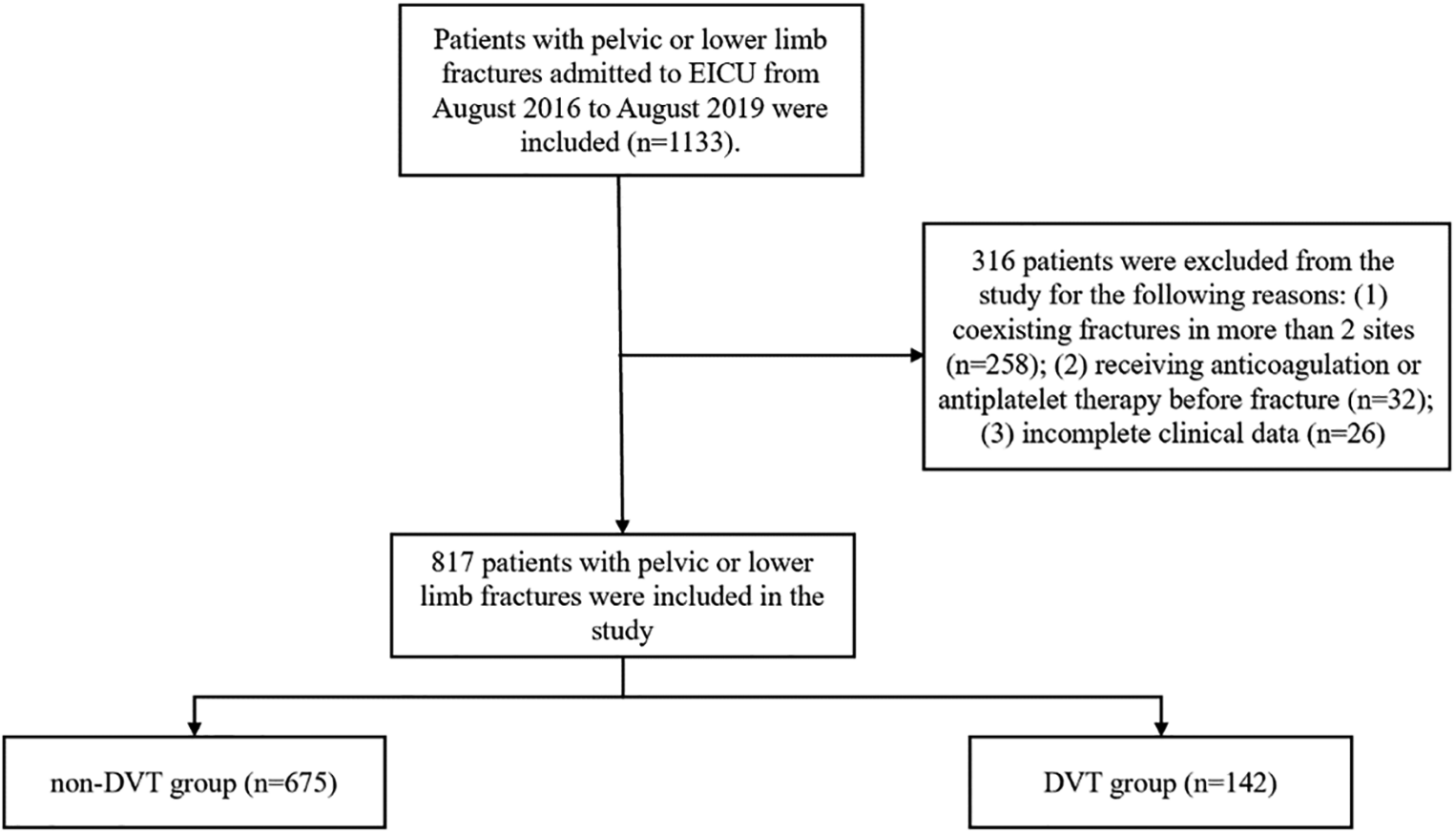
Flow diagram of participants.

The flow diagram of participants is shown in Figure 1. The clinical data of 1,133 patients with pelvic or lower-extremity fractures were analyzed in this study. Of these, 316 patients were excluded, and 817 patients were included in this study, of which 142 (17.38%) had DVT. This study included 368 patients with pelvic fractures, of whom 58 (15.76%) had DVT; of 244 patients with femoral fracture, 66 (27.05%) had DVT; and among 205 patients with tibial fracture, 18 (8.78%) had DVT. Significant differences in multiple injuries, combined brain trauma, fracture site, APACHE II score, ISS score, Autar score, and so forth were found between the DVT and non-DVT groups. The comparison of general participant characteristics between the DVT and non-DVT groups is made in Table 1.

**Table 1:** Comparison of participant characteristics between the DVT and non-DVT groups.

Characteristic	All patients (*n* = 817)	Non-DVT group (*n* = 675)	DVT group (*n* = 142)	*P* value
Age (year)	52.86 ± 17.83	52.33 ± 18.08	55.38 ± 16.41	0.064
Sex (cases, %)	561 (68.67)	468 (69.33)	93 (65.49)	0.370
Risk factors of injury (cases, %)				0.056
Fall	135 (16.52)	110 (16.30)	25 (17.61)	
Fall from height	136 (16.65)	120 (17.78)	16 (11.26)	
Car accident	490 (59.98)	394 (58.37)	96 (67.61)	
Others	56 (6.85)	51 (7.55)	5 (3.52)	
Fracture site (cases, %)				<0.001
Tibial fracture	205 (25.09)	187 (27.70)	18^a^ (12.68)	
Femoral fracture	244 (29.87)	178 (26.37)	66^b^ (46.48)	
Pelvic fracture	368 (45.04)	310 (45.93)	58^a^ (40.84)	
Multiple injuries (cases, %)	383 (46.88)	300 (44.44)	83 (58.45)	0.002
Brain trauma (cases, %)	123 (15.06)	92 (13.63)	31 (21.83)	0.013
Lower-extremity soft-tissue injury and infection (cases, %)	196 (23.99)	167 (24.74)	29 (20.42)	0.273
Hemorrhagic shock (cases, %)	263 (32.19)	212 (31.41)	51 (35.92)	0.296
Sepsis (cases, %)	17 (2.08)	14 (2.07)	3 (2.11)	0.977
APACHE II score	5.54 ± 1.20	5.34 ± 1.14	6.48 ± 1.38	0.003
ISS score	12.55 ± 4.63	12.34 ± 4.57	13.58 ± 4.77	0.004
Autar score	14.38 ± 1.88	14.25 ± 1.86	14.99 ± 1.89	<0.001
GCS score	14.31 ± 1.75	14.34 ± 1.74	14.21 ± 1.80	0.439
HR (beats/min)	92.19 ± 15.80	91.83 ± 15.64	93.88 ± 16.50	0.161
MAP (mm Hg)	80.90 ± 5.82	81.06 ± 5.63	80.11 ± 6.61	0.111
RR (breaths/min)	19.67 ± 3.53	19.60 ± 3.55	19.98 ± 3.45	0.248
PaO_2_/FiO_2_	257.77 ± 48.66	258.74 ± 48.82	253.17 ± 47.77	0.215
pH	7.43 ± 0.07	7.42 ± 0.07	7.43 ± 0.06	0.645
Cr (μmol/L)	82.21 ± 13.76	82.52 ± 14.23	80.72 ± 11.23	0.099
WBC (×10^9^/L)	10.99 ± 3.57	11.04 ± 4.00	10.80 ± 2.68	0.652
Hb (g/L)	97.90 ± 19.22	97.97 ± 19.46	97.54 ± 18.09	0.805
ALT (µ/L)	33.11 ± 8.98	33.11 ± 11.97	33.12 ± 8.07	0.992
AST (µ/L)	29.22 ± 5.49	29.21 ± 4.23	29.30 ± 5.74	0.943
ALB (g/L)	27.05 ± 4.69	27.15 ± 4.81	26.60 ± 4.06	0.160

ALB, albumin; ALT, alanine aminotransferase; AST, acetic transaminase; Cr, serum creatinine concentration; FiO_2_, inspired fraction of O_2_; Hb, hemoglobin; HR, heart rate; MAP, mean arterial pressure; PaO_2_, arterial partial pressure of oxygen; pH, arterial pH; RR, respiratory rate; WBC, white blood cell count.

^a^ ^,^^b^The incidence of DVT in the femoral fracture group was significantly different from that in the tibial fracture and pelvic fracture groups, but no significant difference was found in the incidence of DVT between the tibial fracture and pelvic fracture groups.

### 3.2. Risk factors for DVT in patients with pelvic or lower-extremity fractures

The univariate logistic regression analysis was performed using DVT as the dependent variable and the indicators with statistically significant differences between the DVT and non-DVT groups as the covariates. The results showed that multiple injuries, combined brain trauma, fracture site, APACHE II score, ISS score, Autar score, and so forth might affect the incidence of DVT in patients with pelvic or lower-extremity fractures. The multivariate logistic regression analysis showed that multiple injuries, fracture site, and Autar score were the independent risk factors for DVT in patients with pelvic or lower-extremity fractures in the EICU. The specific results are presented in Table 2.

**Table 2:** Logistic regression analysis results of factors associated with DVT in patients with pelvic or lower-extremity fractures.

Variable	Univariate analysis			Multivariate analysis		
	Crude OR	95% CI	*P* value	Adjusted OR	95% CI	*P* value
Multiple injuries	1.758	1.219–2.537	0.003	2.210	1.166–4.187	0.015
Brain trauma	1.770	1.123–2.789	0.014	1.122	0.508–2.477	0.775
Fracture site			<0.001			<0.001
Femoral fracture^a^	3.852	2.200–6.745	<0.001	4.839	2.688–8.711	<0.001
Pelvic fracture^a^	1.944	1.111–3.400	0.020	2.210	1.225–3.988	0.008
APACHE II score	1.062	1.020–1.016	0.004	0.968	0.896–1.046	0.407
ISS score	1.054	1.017–1.093	0.004	1.015	0.938–1.099	0.710
Autar score	1.239	1.120–1.370	<0.001	1.198	1.061–1.353	0.004

^a^ Statistically obtained OR value relative to the tibial fracture group.

### 3.3. Predictive value of Autar score for DVT in patients with pelvic or lower-extremity fractures

**Figure 2:**
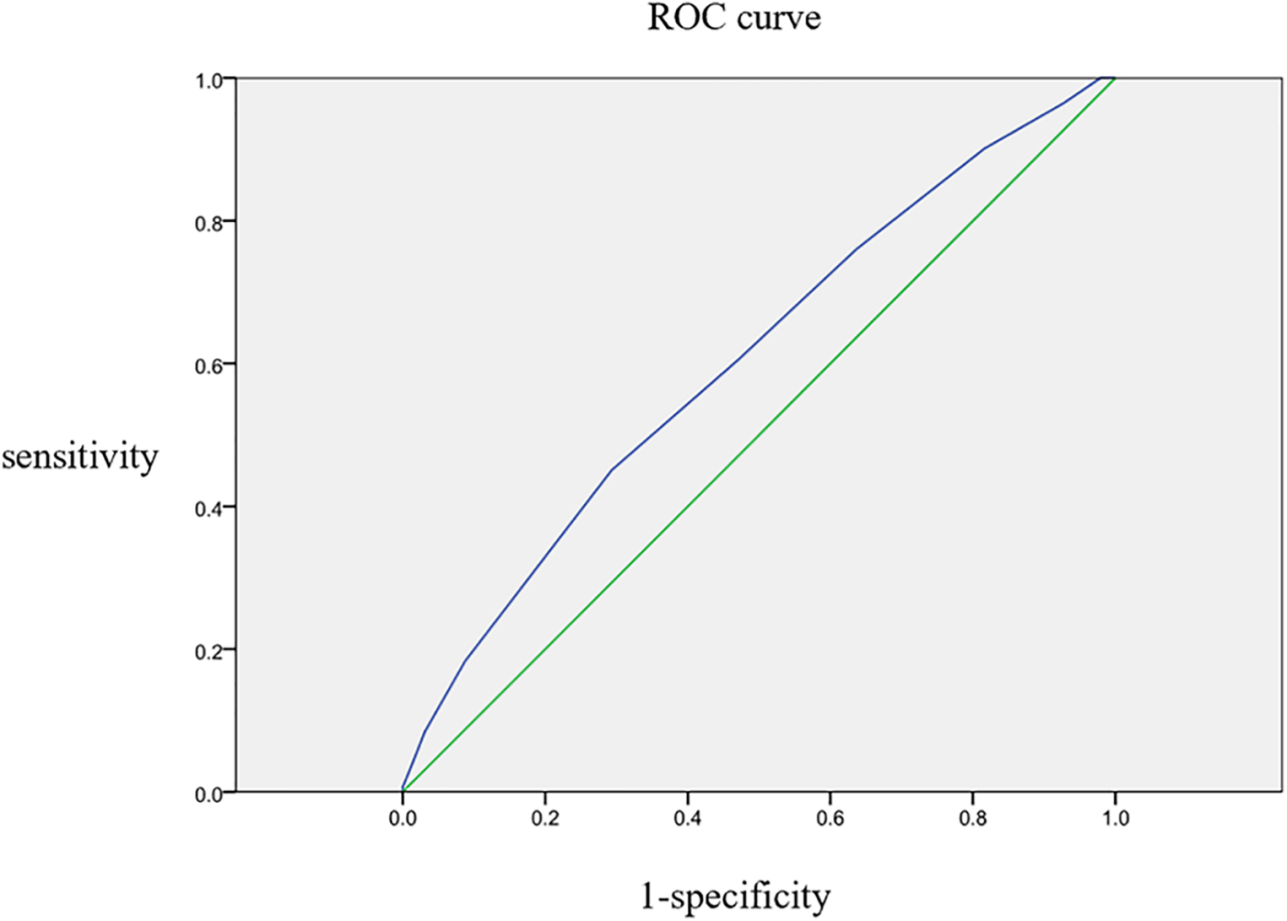
ROC curve of Autar score for predicting DVT in patients with pelvic or lower-extremity fractures.

Autar score was used as the test variable, and the occurrence of DVT in patients with pelvic or lower-extremity fractures was used as the state variable to draw the ROC curve. The results are shown in Figure 2. The analysis of the ROC curve showed that the AUROC was 0.606. When the Autar score was set as the cutoff value of 15.5, the best effect of predicting DVT in patients with pelvic or lower-extremity fractures was achieved, with a sensitivity of 45.1% and a specificity of 70.7%. The specific results are presented in Table 3.

**Table 3:** ROC curve analysis results of Autar score in predicting DVT in patients with pelvic or lower-extremity fractures.

Rating scale	AUROC	95% CI	*P* value	Maximum value of the Youden index	Best cutoff value	Sensitivity (%)	Specificity (%)
Autar score	0.606	0.554–0.657	<0.001	0.158	15.5	45.1	70.7

## 4. Discussion

This study analyzed the clinical data of 817 patients with single-site fractures (pelvic, femoral, or tibial fractures) obtained from the electronic medical records of the hospital information database system. The data were used to compare the incidence of DVT among the three fracture-related groups of patients to detect any statistical significance. At the same time, the chi-square segmentation was used to compare the rate between groups, which is relatively rare worldwide. The results showed that the fracture site was an independent risk factor for DVT in patients with pelvic or lower-extremity fractures, and the incidence of DVT in patients with femoral fractures was significantly higher than that in patients with tibial and pelvic fractures. Femur is the longest and thickest tubular bone in the human body, with high strength and toughness. The force that can cause the fracture of the femur is often stronger than the force that causes the fracture of other parts. The distal femur is a vascular dense area easily involved in trauma and can cause intima injury. After trauma, bed rest and immobilization lead to venous stasis, and edema caused by tissue injury and inflammation can further affect venous drainage. At the same time, the combination of these three factors easily causes the patients with femoral fracture to be at high risk of DVT due to the hypercoagulable state after trauma (8), which thus lead to the highest incidence of DVT in patients with femoral fractures and become an independent risk factor for DVT.

There was a total of 817 subjects in this study, among which 383 (46.88%) had multiple injuries. The total incidence of DVT was 17.38% (142/817), and the incidence of DVT in patients with pelvic fractures was 15.76% (58/368). The incidence of DVT was 27.05% (66/244) in patients with femur fracture and 8.78% (18/205) in patients with tibia fracture. In contrast, the incidence of DVT in patients with non-multiple fractures in our orthopedics department was 8.23% (143/1,737), among which the incidence of DVT in patients with pelvic fracture was 1.81% (4/221), with femoral fracture was 12.22% (113/925), and with tibial fracture was 4.40% (26/591) (21). Patients with pelvic or lower-extremity fractures are at risk of DVT immediately after trauma because of the coexistence of slow blood flow, hypercoagulation, and intimal injury (22). In this study, multiple injuries in patients with fractures were mostly caused by high-energy wounds such as traffic accidents and falling from high altitudes. The external energy leading to injury is usually greater and the intima injury, blood stasis, and coagulation disorder are often more severe in patients with multiple injuries than in those without multiple injuries (23). At the same time, hemorrhagic shock, hypoperfusion, and ischemia–reperfusion injury after fluid resuscitation in patients with multiple injuries may make the conditions more serious, leading to multiple injuries as one of the independent risk factors for DVT in patients with pelvic or femur fractures (24). Despite the preventive measures taken, the incidence of DVT in patients with multiple injuries is still high (25). Thus, for emergency physicians in EICU to treat patients with multiple injuries accompanying lower extremity fracture, they should raise awareness of thrombosis treatment. On the other hand, although no subvariables constituting polytrauma were found to be independent risk factors for DVT, polytrauma still had a significant impact on the occurrence of DVT in patients with fractures.

The first version of the Autar scale was developed by Ricky Autar in 1994, which was used to guide medical staff to identify high-risk groups of DVT in clinical practice. The latest version of the Autar scale was revised in 2002 (19). The DVT risk of patients was evaluated from seven aspects: age (0–5), body mass index (0–4), exercise ability (0–4), trauma (1–4), special risk (1–4), surgical risk (1–4), and high-risk underlying diseases (1–7). The total score of each item was added up. The total score of ≥15 was categorized as the high-risk group, score of 11–14 as the medium-risk group, and score of ≤10 as the low-risk group. Pelvic and lower-limb trauma, surgery below the waist, bed rest, and other items with high scores in the Autar scale seem to be specific for assessing the risk of DVT in patients with fractures. Therefore, this study used it as a predictive tool for DVT in patients with pelvic or lower-extremity fractures. This study analyzed the clinical data of 817 patients. Also, using the Autar scale to evaluate the predictive value of DVT in patients with pelvic or lower-extremity fractures is novel. The Autar scale method is relatively simple and convenient for clinical staff to use, but its value in predicting DVT in patients with fractures needs further research, and it may be necessary to increase the score of the diagnostic cut-off value to increase the sensitivity of predicting DVT.

In general, an AUROC of 0.5 indicates no diagnostic value, 0.5 < AUROC < 0.7 indicates poor diagnostic accuracy, 0.7 ≤ AUROC < 0.8 indicates acceptable diagnostic accuracy, 0.8 ≤ AUROC < 0.9 indicates good diagnostic accuracy, and AUROC ≥ 0.9 indicates excellent diagnostic accuracy (26). In this study, the AUROC of Autar score as a diagnostic tool to predict DVT was 0.606, indicating its poor accuracy in predicting DVT in patients with pelvic or lower-extremity fractures. The Youden index, also known as the correct index (27), is equal to the sum of sensitivity and specificity minus 1 and represents the total ability of the screening method to detect true positives and true negatives. The value of the covariate corresponding to the maximum value of the Youden index can be used as the diagnostic critical value, namely the cutoff value (28). In this study, when the cutoff value of the Autar score was 15.5, the comprehensive value of predicting DVT in patients with pelvic or lower-extremity fracture was the highest. Also, the specificity was 70.7%, but the sensitivity was only 45.1%, reflecting the low predictive value of the Autar score for DVT in patients with pelvic or lower-extremity fractures. Autor et al. showed that the AUROC of this scoring method for predicting the occurrence of DVT in the study object was 0.696. When the cutoff value was 11, the sensitivity of predicting the occurrence of DVT in the objective participants was about 70% (19), and the result was also not ideal. However, the Autar scoring method is relatively simple and is beneficial for clinicians and nurses to use, but its value in predicting DVT in patients with fractures needs further research, and it may be necessary to increase the score of the diagnostic cut-off value to increase the sensitivity of predicting DVT.

The fracture itself is a high-risk factor for DVT (29), and patients with a femoral fracture complicated with multiple injuries have a higher risk of DVT. The DVT before surgery may fall off during the surgery and lead to fatal pulmonary embolism, and hence its needs attention. For patients with pelvic or lower-extremity fractures, DVT preventive measures should be taken in the case of no contraindications. If patients still have proximal DVT (including iliac vein, femoral vein, superficial femoral vein, and popliteal vein thrombosis) (30), an inferior vena cava filter should be placed before the surgery to prevent fatal pulmonary embolism.

This study still had some limitations. First, this was a single-center retrospective case control study, and the number of patients included in the study was small. The factors affecting the prognosis of patients might not have been fully considered, inevitably leading to bias. Further confirmation of findings through multicenter, large-sample, and prospective studies is needed. Next, although vascular ultrasound has gradually replaced venous angiography and is widely used, it is not the “gold standard.” Therefore, the possibility of a false positive or false negative in some DVT diagnoses was not excluded in this study.

## Data Availability

The raw data supporting the conclusions of this article will be made available by the authors, without undue reservation.
